# Supplementary material for: Are carbon emissions trading and green financial instruments synergistic? -Comprehensive quantitative research based on content analysis
Source: PLoS One. 2024 Mar 7;19(3):e0298601. doi: 10.1371/journal.pone.0298601 (PMC10919601; doi:10.1371/journal.pone.0298601)
Supplement: S1 Data — (ZIP) [file pone.0298601.s001.zip › Data/地级市Z_F工作报告.zip!/╣π╬≈/║╪╓▌╩╨/└┤╫╘╣1⁄2╓┌║┼í╛┬φ┐╦╩2╛▌═°í┐_2019.docx]

一、2018年工作回顾

　　过去一年,是进入新常态以来贺州发展最好的一年。面对严峻复杂的经济形势,在自治区党委、政府和市委的坚强领导下,在市人大、市政协的监督支持下,我们坚持以习近平新时代中国特色社会主义思想为指导,深入贯彻党的十九大、十九届二中、三中全会精神,牢固树立“四个意识”,坚定“四个自信”,坚决做到“两个维护”,紧紧围绕“三大定位”新使命和“五个扎实”新要求,以改革开放40周年和自治区成立60周年为契机,聚焦市人大四届三次会议确定的目标任务,全面开展“实体经济服务年”“作风建设年”活动,加快培育“五大新引擎”,大力弘扬“说干就干,干就干好”的工作作风,团结一心、克难攻坚,推动全市经济在艰难形势下打开新局面,呈现速度加快、结构转优、业态向新的良好态势,社会实现和谐稳定发展。

　　——经济发展增速首次跃升全区第一方阵。初步统计,全市完成地区生产总值601亿元,同比增长9.3%；人均GDP为2.92万元,提升了两位。其中一、二、三次产业增加值分别完成122亿元、209亿元、270亿元,分别增长4.5%、11.2%、10%。规上工业总产值增长30%,增速领跑全区。全市财政收入完成58.16亿元,增长9.5%,总量赶超两市。非税收入占一般公共预算收入比重下降3.9个百分点,收入质量明显提高。人民币各项贷款增长12.25%,存贷比为66.83%。社会消费品零售总额增长11.5%。GDP、工业增加值、服务业增加值、财政收入等经济指标的增速均排全区前列,我市首次在全区经济工作会议上作典型发言,摆脱了长期处于全区排名靠后的尴尬局面。

　　——发展战略地位从此由边缘走向前沿。去年,自治区党委主要领导到贺州调研时,提出了“发挥优势、突出特色、全力东融、加快发展”的总体要求,赋予贺州创建广西东融先行示范区的新定位。我市围绕全力东融,编制实施创建广西东融先行示范区工作方案、发展规划和三年行动计划,成立驻粤港澳大湾区经贸联络处,设立深圳贺江新产业育成中心,组建东融产业投资集团,将东融触角向深圳、广州一线城市延伸,为全力东融布好局、搭好台、起好步,抓住了建市以来最大、最好的发展机遇,摆脱了长期处于开放合作边缘的被动局面。

　　——产业发展布局加速转向新兴产业引领。设立贺州新经济新业态发展基金(母基金),启动广西数字贺州产业园、贺州健康云港产业园、广西倍易通电子产业园等三大新兴产业园规划建设；集聚了广西软视科技、广西一曜生物、双展生物、普唯尔生命科技等20多个新经济新业态项目,泰达新源股权投资、海德创业投资等企业总部纷纷落户贺州；全市战略性新兴企业53家,产值64亿元,新旧动能加快转换,努力实现产业“换道超车”,摆脱了长期处于传统产业主导的落后局面。

　　——人民群众的获得感、幸福感、安全感大幅提升。动车站完成升级改造,实现人车分流,候车大厅更亮堂、更大方,让市民出行更舒适、更便捷；公交在线查询、扫码支付,街区停车智能管理,车管业务网上办理,让市民的日常生活更时尚、更方便；新区高楼拔地而起,城市夜景美轮美奂,东融大道、潇贺大道等市政道路的提质,东融山庄等商务平台的打造,让“寿城”门户更亮丽、更大气；爱莲湖公园、大钟山公园等四大公园品质不断提升,文化中心活动日益丰富,让市民的休闲生活更加精彩、更有品味；启用市民服务中心,推进“一事通办”“一网通办”改革,让市民办事、企业办证变得更顺畅、更舒心；开展生态环境整治专项行动,让扬尘降、街道亮,市民心情更舒畅；“扫黑除恶”专项行动强势推进,群众安全感节节攀升,排名全区领先。

　　一年来,我们主要做好了以下工作:

　　一是打好“五场硬仗”,脱贫攻坚取得新成效。有望实现富川瑶族自治县和93个贫困村摘帽、7.65万贫困人口脱贫,全市贫困发生率降至4.7%。落实财政扶贫资金18.89亿元,整合涉农资金10.21亿元,全力支持扶贫攻坚。投入产业奖补资金0.9亿元,脐橙、贡柑、茶叶、食用菌、蔬菜等特色产业在贫困地区拔节生长,贫困村特色产业覆盖率达84.84%。贫困地区基础设施建设投入5.1亿元,修建村屯道路项目1040个866公里、危改项目5814户,解决安全饮水问题2.25万户、用电问题8299户。盘活村集体资产,全市所有行政村集体经济收入达到2万元以上,已脱贫和计划脱贫摘帽的93个贫困村集体经济收入均达3万元以上。协调落实2.79亿元,支持平桂区“土瑶”聚居深度贫困村脱贫攻坚。完成26个易地扶贫搬迁安置点建设,搬迁入住6.16万人。落实粤桂扶贫协作资金7260万元,推进帮扶项目建设,肇庆市鼎湖区56个单位企业与昭平县57个贫困村结对帮扶,四会市与富川瑶族自治县共建扶贫协作产业园。加大教育、健康扶贫等工作力度,共资助家庭经济困难学生12.88万人次,发放国家助学金、困难补助金等1.02亿元,贫困人口大病住院报销比例达91.27%。

　　二是加快动能转换,实体经济发展步入新轨道。经济结构协调性增强,三次产业结构从2017年的21.1:38.4:40.5优化为20.3:34.8:44.9。现代服务业发展动力强劲,新增上规上限服务业企业60家,服务业税收占税收总收入的55.2%,对GDP增长的贡献率达46%。姑婆山温泉足球小镇等大旅游、大健康项目建设提速,姑婆山产业区入选广西养生养老小镇。大皇蜂无车承运等项目落户贺州,为培育大物流搭建了信息平台。“长寿?养生”经济高速发展,康养产业项目累计完成投资约66亿元,“梦里黄姚”、黄姚?龙门街等旅游项目开工建设,昭平县成功创建广西特色旅游名县,富川福利国际慢城成为自治区生态旅游示范区,新增国家4Ａ级旅游景区3家,旅游总接待人数3125万人次,旅游总消费394亿元,分别增长41%和44%。工业发展加快转型升级,规上工业增加值增长18%,钢铁行业部分恢复生产,产值增长149.6%。新增规上碳酸钙企业17家,碳酸钙行业规上工业总产值增长25%。旺高工业区跻身中国建材园区20强。全市6个装配式建筑产业基地实现投产,13个装配式建筑试点项目开工建设,广西装配式建筑现场观摩会在我市召开。建筑产业异军突起,建筑业总产值34亿元,增长32%,房地产销售面积154万平方米,增长25%,销售额76亿元,增长40.7%。农业产业化特色鲜明,创建国家有机产品认证示范县在全区率先实现全覆盖,新增3个自治区级核心示范区、12个县级示范区,“富川脐橙”通过国家级农产品地理标志示范样板验收,6家专业合作社上榜国家农民合作示范社,建成长寿食品一条街。粮食产量71.88万吨,农林牧渔业总产值增长4.6%。成功在广州举办农业产业招商会,签约项目10个,总投资7.45亿元,农业东融再迈新步伐。另外,首创设立非公经济发展服务中心,非公经济对全市经济增长的贡献率超过60%。

　　三是扩大开放合作,全力东融打开新局面。深入实施产业东融、人才东融、旅游东融、康养东融等“五大行动”,开辟东融“快车道”。推进产业东融,将工业园区和产业区作为全力东融的主阵地,创新规划招商、资本招商、孵化招商,主动承接粤港澳大湾区产业转移,积极探索区域经济融合发展的新路子。招商引资到位资金470亿元,增长13.6%,其中来自粤港澳大湾区的投资占比达64.8%；与浪潮集团、香港锦绣集团等5家知名企业签订项目合作协议,总投资超过150亿元,产业布局更加多元。贺州海关组建完成,结束了我市一直以来没有海关的历史。推进旅游东融和康养东融,积极创建国家全域旅游示范市,主动拓展大湾区客源市场,来自大湾区的游客比重达70%。建成了康养护理示范基地“贺寿园”,引进了力达新医药港等健康产业项目,国际健康长寿论坛成功举办,并永久落户贺州,“生态贺州?长寿胜地”和“住几天管用”品牌得到高度关注和广泛传播。推进人才东融,先后与广东财经大学、暨南大学、广州中医药大学、深圳大学等一批高校签订市校合作协议,成功举办中国创新设计大会贺州峰会暨广西东融先行示范区产业合作创新发展论坛、粤港澳大湾区高层次人才寿城行等活动。

　　四是扩大有效投资,重大项目建设实现新突破。建立贺州市重大项目调度会议制度,推行市厅级领导联系服务项目机制,统筹推进重大项目468个,完成投资196亿元。中央预算内投资项目完成率荣登全区高质量发展红榜。贺富一级路贺街至望高段建成通车,贺巴高速贺州段等项目加快建设,贺连高速广西段、信梧高速等一批重大基础设施项目开工建设,广西大健康产业检验检测认证中心、日轻高纯铝、续宝矿业等一批产业项目加快推进,贺州第二高级中学扩建工程、平桂文化体育中心等一批民生项目实现竣工。全市园区基础设施项目投入30亿元,建成园区路网50公里、标准厂房20万平方米。着力破解项目用地难题,完成征地9194.29亩,拆除房屋735间,新增建设用地指标1.26万亩,盘活存量建设用地2.15万亩。打破项目融资瓶颈,获得自治区转贷新增债券26.94亿元,争取上级各类专项资金48.1亿元,实现银行融资45.28亿元；采用PPP模式融资,获得亚行、世行、欧佩克、法开署贷款支持,项目总投资约70亿元,有力保障了项目资金需求。

　　五是推动创新发展,深化改革迈出新步伐。国企“混改”释放活力,促成了农投集团与天贺正丰公司、交投集团与广西鑫柏轩集团、城投集团与通号集团资本合作,特别是促成正润集团与广投集团的战略合作,不但化解了正润集团的债务危机,而且推动了铝电子产业转型升级,还筹措了新经济新业态产业发展资金。国有企业在融措建设资金、完善基础设施、推动经济增长等方面发挥重要作用,监管企业营业收入136.18亿元,增长22.81%。园区体制机制改革激发动力,实施公开竞聘上岗、改革薪酬分配和绩效考评等举措,园区提速发展,五大工业园区实现规上工业总产值280亿元,增长45%以上,入库税收超10亿元。农村综合改革全面展开,农村土地承包经营权确权登记颁证工作基本完成,国有林场改革顺利通过自治区验收,农村集体产权制度改革和“三权分置”、小型水利工程管理体制改革稳步推进。“放管服”改革持续深化,审批事项网上申报率91.27%,承诺件办理提速76.4%,工商注册登记、施工许可证办结压缩至2天以内。全面落实“三去一降一补”政策措施,累计为企业节约成本19亿元。市本级企事业单位公车改革圆满完成。

　　六是统筹城乡建设,生态贺州呈现新面貌。城市综合品质全面提升,《贺州市城市总体规划(2016-2035年)》获自治区人民政府批复,《贺州市中心城区风貌规划导则》等多个专项规划编制完成,城市规划面积从78平方公里扩大到129.83平方公里。城市品质提升三年行动深入实施,统筹推进灵峰山以西旧住宅区综合整治改造工程和城东新区基础设施建设,贺州果蔬批发市场、八步中心市场升级改造等项目投入使用。城市建成区面积扩展到53.43平方公里,城镇化率达46.8%。“美丽贺州?宜居乡村”建设持续推进,八步区善华村等6个村入围“美丽广西”乡村建设示范村,钟山县龙道村等7个村入选第七批中国历史文化名村,富川瑶族自治县岔山村等10个村入选第五批中国传统村落名录,农村新型生态社区建设经验在全区、全国推广,全区城乡建设用地增减挂钩助力脱贫攻坚和乡村振兴现场会在贺州召开。县域经济发展齐头并进,八步区经受住了“去产能”的考验,现代服务业快速发展,多项主要经济指标总量、增幅在全市领先；平桂区推动新城建设、园区发展和产业转型升级成绩喜人；钟山县探索出了一条以投资促建设、以产业换发展的创新之路,新型城镇化步伐加快；富川瑶族自治县在集中力量打好脱贫攻坚战的同时,乡村旅游和特色农业亮点纷呈；昭平县打造旅游、茶叶、健康饮用水等生态特色产业成效初显。生态优势不断巩固,全面打响水、大气和土壤污染防治攻坚战,持续抓好中央、自治区环境保护督察组反馈问题整改工作。世行贷款贺州市水环境治理与城市综合发展项目加快实施。大气污染防治“百日攻坚”行动卓有成效,城区环境可吸入细颗粒物(PM2.5)年均浓度为37.95微克/立方米,下降10.2%,完成自治区下达的年度目标任务。饮用水水质达标率100%,富川龟石水库成为国家湿地公园,我市生态保护与建设示范区入选国家典型示范区。

　　七是保障改善民生,社会事业迈上新台阶。全市民生支出156.03亿元,占一般公共预算支出的80.1%。科教事业有新进步,获得自治区创新驱动资金支持4000万元。投入资金3.19亿元用于新改扩建中小学校(幼儿园)145所,新增学位8364个,新增普惠性幼儿园36所,中心城区8所中小学校项目陆续开工建设,投入资金10.03亿元实施“全面改薄”“普通高中攻坚计划”。钟山县、富川瑶族自治县、八步区、昭平县先后通过义务教育基本均衡发展国家督导评估。文化体育事业取得新成绩,参与央视《魅力中国城》第二季竞演,获得“优秀魅力城市”称号,黄姚古镇被评为“年度魅力小镇”。成功举办第四届中国(贺州)新媒体群英会暨广西贺州长寿文化节以及中国-东盟(富川)国际龙狮争霸赛、中国龙舟赛(广西?昭平站)、贺州市第一届“东融杯”青少年足球锦标赛等赛事。社会保障更加有力,超额完成五险扩面征缴,城镇新增就业1.11万人,城镇登记失业率控制在2.39%以内。建成棚户区改造住房1.28万套,累计分配入住公租房1.7万套。发放城乡低保金3.24亿元,惠及城乡困难群众11.3万人。投入1.1亿元实施农村“一事一议”、危房改造、道路硬化等工程。医保“一门式”系统上线,实现区内外大病保险直报,全市二级以上定点医疗机构基本实现大病保险“一站式”即时结算,争取中央和自治区资金2.35亿元,扩建2个县级医院和36个乡镇卫生院。居民消费价格指数(CPI)涨幅控制在2.4%以内,物价总体平稳。社会大局和谐稳定,创建自治区双拥模范城、广西食品安全示范城市通过自治区现场考评。扎实推进全国民族团结进步示范市创建工作,成功举办第十四届中国瑶族盘王节、桂台客家宗亲经济文化论坛。“平安贺州”“法治贺州”“信用贺州”建设成效明显,城区“两抢”零报警天数357天,全市刑事案件同比下降7.52%,圆满完成自治区成立60周年大庆安保维稳等各项工作。

二、2019年工作安排

　　今年是新中国成立70周年,是同步全面建成小康社会、实施“十三五”规划的关键一年,是全区全面贯彻落实习近平总书记重要题词精神的第一年,也是我市创建广西东融先行示范区的第一年。做好今年的工作意义重大、影响深远。

　　2019年政府工作的总体要求是:以习近平新时代中国特色社会主义思想为指导,全面贯彻党的十九大和十九届二中、三中全会精神以及中央庆祝改革开放40周年大会,中央、自治区经济工作会议精神,持续深入落实“三大定位”新使命和“五个扎实”新要求,统筹推进“五位一体”总体布局,协调推进“四个全面”战略布局,坚持稳中求进工作总基调,坚持新发展理念,坚持推动高质量发展,坚持以供给侧结构性改革为主线,坚持深化市场化改革、扩大高水平开放,统筹推进稳增长、促改革、调结构、惠民生、防风险工作,进一步稳就业、稳金融、稳外贸、稳外资、稳投资、稳预期,紧紧围绕“发挥优势、突出特色、全力东融、加快发展”总体要求,始终以东融统领工作全局,继续打好三大攻坚战,奋力赶超跨越、打造广西东融先行示范区,推动新经济新业态新产业集群发展,着力提升开放水平,着力壮大实体经济,着力推动项目建设,着力深化重点改革,着力统筹城乡发展,着力抓好民生建设,促进经济社会持续健康发展,为同步全面建成小康社会打下决定性基础,努力为“建设壮美广西　共圆复兴梦想”贡献贺州力量,以优异的成绩庆祝中华人民共和国成立70周年。

　　今年经济社会发展的主要预期目标是:地区生产总值增长8%,财政收入增长6%,规模以上工业增加值增长8%,固定资产投资增长12%,社会消费品零售总额增长10.5%,外贸进出口总额增长10%；居民消费价格涨幅控制在3.5%以内,城镇居民人均可支配收入和农村居民人均可支配收入分别增长7%和10%,城镇登记失业率控制在4.2%以内,常住人口城镇化率达48%,节能减排降碳指标完成自治区下达任务。

　　今年,我们将重点抓好以下工作:

　　(一)统筹平台建设与产业融合,全力创建广西东融先行示范区。

　　(二)统筹精准脱贫与防范返贫,坚决打赢脱贫攻坚战。

　　(三)统筹优化存量与培植增量,着力壮大实体经济。

　　(四)统筹有效投资与要素保障,强力推进重大项目建设。

　　(五)统筹国企改革与园区改革,全面优化营商环境。

　　(六)统筹城市提质与乡村振兴,扎实推进城乡区域协调发展。

　　(七)统筹民生保障与社会治理,不断提升公共服务水平。

三、全面加强政府自身建设

　　做好今年的各项工作,必须进一步加强政府自身建设,提高新时代推动贺州高质量发展的能力和水平,提升政府的公信力和执行力,努力做到以人民为中心,为人民服务,对人民负责,受人民监督,打造人民满意的政府。

　　(一)推动政府职能转变。推动市直各部门对本行业权责清单进行调整和规范,大幅减少政府资源的直接配置,强化事前事中事后监管,凡是市场能自主调节的就让市场来调节,凡是企业能干的就让企业来干。严格控制“三公”经费支出,在保工资、保运转、保基本民生的基础上,确保做到少投入、多产出,少用钱、多办事,少用人、多干活,提高政府行政效能。推动决策、执行、管理、服务和结果“五公开”,及时回应群众关心关注的问题。进一步完善考核激励机制,强化责任担当和督查问责,推动工作落实。全面完成政府机构改革,确保机构、职责和队伍等按要求及时调整到位,建立协调有序高效的政务运行机制。

　　(二)推进法治政府建设。坚决服从市委领导,落实“三重一大”事项报告制度,构建决策科学、执行坚决、监督有力的权力运行机制。自觉接受人大及其常委会监督,认真落实各项决议、决定并定期报告工作,自觉接受人民政协的民主监督,接受社会舆论监督,办理好人大代表建议和政协委员提案,虚心听取民主党派、工商联、人民团体和各界人士的意见建议。构建覆盖城乡居民的公共法律服务体系,严格规范公正文明执法。推进全民普法,增强政府工作人员法治思维,提高依法行政水平。

　　(三)加强干部队伍建设。牢固树立“担当为要、实干为本、发展为重、奋斗为荣”的为政理念,大力弘扬“说干就干、干就干好”的工作作风,塑造“专业干事、专心做事、专注成事”的干部形象,铸就“忠诚、干净、担当、专业、勤恳”的政治品格。认真贯彻落实《关于进一步激励广大干部新时代新担当新作为若干措施》,旗帜鲜明地保护敢于担当、勇挑重担、抓铁有痕的干部,树立让吃苦者吃香、有为者有位、为担当者担当的用人导向,大胆使用能干事、想干事、干成事的干部,有能力者给空间,有技术者给舞台。坚决调整那些表态多、调门高、行动少、落实差、不作为、慢作为的干部。加强新时代干部队伍专业化建设,激励干部牢牢盯住重点工作,建设一个项目,培养一个团队,带出一批干部,成为经济工作的行家里手。突出事业为上,坚持一把尺子量到底,一张蓝图绘到底,一个目标干到底,在推动经济高质量发展这场“大考”中考出好成绩,练就真本领。

　　(四)加强党风廉政建设。贯彻落实中央“八项规定”精神和国务院“约法三章”,严格履行党风廉政建设“一岗双责”,执行《关于新形势下党内政治生活的若干准则》和《中国共产党党内监督条例》,筑牢拒腐防变的思想防线和制度防线。坚持无禁区、全覆盖、零容忍,保持惩治腐败高压态势,充分发挥审计监督检查作用,严格依照纪律和法律的尺度,强化日常管理和监督,营造干净干事的氛围,保证干部清正、政府清廉、政治清明。

　　各位代表!蓝图已经绘就,机遇就在眼前,发展时不我待。新时代新征程,需要新担当新作为,让我们更加紧密团结在以习近平同志为核心的党中央周围,高举习近平新时代中国特色社会主义思想伟大旗帜,在自治区党委、政府和市委的正确领导下,发挥优势、突出特色、全力东融、加快发展,大力弘扬“说干就干、干就干好”的工作作风,为“建设壮美广西、共圆复兴梦想”贡献贺州力量,为决胜同步全面建成小康社会、开创贺州高质量发展新局面而努力奋斗!
